# Beyond BMI: visceral adiposity assessed by the lipid accumulation product index shows independent associations with IL-5 and TNF-β in older adults

**DOI:** 10.1007/s40520-026-03338-y

**Published:** 2026-02-24

**Authors:** Francesca Mancinetti, Patrizia Bastiani, Martina Alunno, Veronica Tecchio, Michela Scamosci, Roberta Cecchetti, Patrizia Mecocci, Virginia Boccardi

**Affiliations:** 1 Division of Gerontology and Geriatrics, Department of Medicine and Surgery, University of Perugia-Santa Maria della Misericordia Hospital, Perugia, Italy; 2 Division of Clinical Geriatrics, NVS Department, Karolinska Institutet, Stockholm, Sweden

**Keywords:** Aging, Cytokines, Inflammaging, Metabolism, Older adults, Visceral adiposity

## Abstract

**Background:**

Visceral adiposity contributes to inflammaging, but body mass index (BMI) may poorly reflect fat redistribution in late life. The lipid accumulation product (LAP) index is a surrogate of visceral adiposity; its immune correlates in older adults remain incompletely defined.

**Methods:**

In this cross-sectional study, we analyzed 206 community-dwelling older adults (mean age 75.9 ± 7.5 years; 57.3% women). Clinical, functional, anthropometric, metabolic, and biochemical data were collected with circulating cytokine and chemokine concentrations. Associations between LAP and immune mediators were explored using correlation analyses and progressively adjusted multivariable linear regression models.

**Results:**

LAP did not differ by sex (*p* = 0.825). Women showed lower IL-10 (*p* = 0.011), IL-12p70 (*p* = 0.004), IL-3 (*p* = 0.017), IL-4 (*p* = 0.006), IL-15 (*p* = 0.013), and higher RANTES/CCL5 (*p* = 0.029). In the overall cohort, LAP correlated with BMI (*p* < 0.001) and glycaemia (*p* < 0.001), and inversely with HDL-C (*p* < 0.001). LAP was correlated with eotaxin (*p* = 0.029), MCP-1 (*p* = 0.038), IL-5 (*p* = 0.016), and TNF-β (*p* = 0.007) after age/sex adjustment. In fully adjusted regression models, IL-5 (*p* = 0.023) and TNF-β (*p* = 0.002) remained independently inversely associated with LAP, whereas MCP-1 and eotaxin lost significance after BMI adjustment. Associations between IL-5, TNF-β and other conventional measure of central adiposity ( waist-to-hip ratio) were weaker and less consistent than those observed for LAP index.

**Conclusion:**

In older adults, LAP index may capture an immunometabolic phenotype not fully explained by BMI alone. Higher LAP index is independently associated with lower IL-5 and TNF-β, suggesting a potential selective impairment of regulatory immune signaling with increasing visceral lipid accumulation.

**Supplementary Information:**

The online version contains supplementary material available at 10.1007/s40520-026-03338-y.

## Introduction

Aging is characterized by a chronic, low-grade inflammatory state commonly referred to as inflammaging [1], which is increasingly recognized as a central biological mechanism underlying the development of multiple age-related conditions, including cardiovascular disease, type 2 diabetes, sarcopenia, and cognitive impairment [2, 3]. This persistent inflammatory milieu emerges from the progressive remodelling of the immune system, metabolic dysregulation, and age-related tissue changes, and contributes to frailty, reduced physiological reserve, and vulnerability to stressors in later life [4].

Among the factors shaping immune modulation in older adults, adipose tissue—and particularly visceral adiposity—plays a pivotal role [5]. Beyond its function as an energy reservoir, visceral fat acts as an active endocrine and immunomodulatory organ, secreting a broad spectrum of cytokines, chemokines, and adipokines that influence both metabolic and immune homeostasis [5, 6]. Excess visceral fat accumulation has been consistently associated with elevated circulating levels of pro-inflammatory mediators, such as tumor necrosis factor-α (TNF-α) and interleukin-6 (IL-6) [7], as well as chemokines involved in immune cell recruitment, including monocyte chemoattractant protein-1 (MCP-1) [8]. These alterations may contribute to insulin resistance, endothelial dysfunction, and heightened cardiometabolic risk, processes that are particularly relevant in aging populations [8, 9].

Traditional anthropometric indices, most notably body mass index (BMI), have important limitations in capturing age-related changes in body composition and fat distribution. In older adults, BMI may fail to reflect visceral adiposity accurately due to concomitant loss of lean mass, changes in fat redistribution, and sex-specific trajectories of aging [10]. Other anthropometric measures, such as waist circumference and waist-to-hip ratio (WHR), are widely applied to characterize overall and central adiposity. Nevertheless, these metrics provide a limited representation of body fat biology, as they do not differentiate between different adipose tissue compartments and may overlook clinically relevant features of fat distribution [11, 12]. Although these measures remain useful in cardiometabolic risk stratification [13], they frequently inadequately reflect the substantial metabolic variability observed among individuals sharing comparable BMI values. In this context, the Lipid Accumulation Product (LAP) index—derived from waist circumference and fasting triglycerides—has emerged as a simple and validated surrogate marker of visceral adiposity, metabolic risk [14] and sarcopenia in adults [15]. LAP index has been shown to outperform BMI in predicting insulin resistance, metabolic syndrome, and cardiovascular outcomes across different populations [14, 16], including older individuals [17]. By integrating an anthropometric component with circulating lipid burden, LAP may therefore better capture the biological consequences of visceral fat accumulation than anthropometric measures alone, particularly in late life.

Despite growing evidence supporting the clinical relevance of LAP, little is known about its immunological correlates in aging. In particular, the extent to which LAP is associated with specific circulating cytokine and chemokine profiles and whether these associations differ from those observed with conventional anthropometric measures of central adiposity, remains incompletely defined. Addressing these gaps may provide insight into the immunometabolic pathways linking visceral adiposity to inflammaging and age-related vulnerability. Therefore, the present study aimed to characterize the associations between LAP and circulating immune mediators, with a specific focus on cytokines and chemokines implicated in immune regulation, chemotaxis, and inflammatory signaling, in a cohort of community-dwelling older adults.

## Methods

### Study design and participants

This study is a cross-sectional analysis of data derived from a clinical research initiative at the University of Perugia, as previously reported [18]. Participants were community-living older outpatients referred for comprehensive geriatric and cognitive evaluation. Participants were recruited between January 2016 and December 2024. Of the 1,731 participants initially screened, 206 met all eligibility criteria and were included in the final analysis: exclusion criteria included clinical or biological evidence of acute or chronic inflammatory or infectious diseases, malignant neoplasms, autoimmune or hematological disorders, diabetes mellitus, or treatment with anti-inflammatory drugs; inclusion criteria comprised individuals aged ≥ 65 years who were able to provide written informed consent. All participants provided informed consent prior to enrolment. The study was conducted in accordance with the principles of the Declaration of Helsinki and approved by the Regional Ethics Committee (Protocol No. 8005/16/ON).

### Multidimensional geriatric assessment

Participants underwent a standardized multidimensional geriatric assessment by trained clinicians [18]. Demographic, clinical, pharmacological, and functional data were collected through structured interviews and validated scales. Functional status: assessed using the Basic Activities of Daily Living (BADL) [19] and Instrumental Activities of Daily Living (IADL) [20] scales. Cognitive function: evaluated with the Mini-Mental State Examination (MMSE) [21]; scores range from 0 to 30, with values < 24 indicating cognitive impairment. Mood assessment: depressive symptoms were assessed with the 15-item Geriatric Depression Scale (GDS) [22], with scores categorized as: 0–4 no depression, 5–8 mild, 9–11 moderate, and 12–15 severe depressive symptoms. Nutritional status: evaluated with the Mini Nutritional Assessment (MNA) [23]; scores < 17 indicate malnutrition, 17–23.5 risk of malnutrition, and ≥ 24 normal nutritional status. Multimorbidity: quantified with the Cumulative Illness Rating Scale–Geriatrics (CIRS-G) [24], which assesses impairment across 14 organ systems on a scale from 0 (no impairment) to 4 (life-threatening). The CIRS-G total score reflects global morbidity, while the Severity Index and Comorbidity Index provide complementary measures of disease burden.

### Clinical and biochemical measurements

Anthropometric measurements were obtained using standardized procedures. Weight and height were measured, respectively, and BMI was calculated as weight (kg) divided by height squared (m²). Waist and hip circumferences were measured with a flexible tape, and the WHR was calculated. Blood pressure was measured in the seated position using the Riva-Rocci method after 5 min of rest. Venous blood samples were collected after an overnight fast. Serum glucose and lipid profiles (total cholesterol, HDL-C, triglycerides) were analyzed using enzymatic methods; LDL-C was calculated using the Friedewald formula. The LAP index, a surrogate marker of visceral adiposity, was calculated as follows:

- For women: (waist circumference [cm] − 58) × triglycerides [mmol/L].
- For men: (waist circumference [cm] − 65) × triglycerides [mmol/L].

Triglycerides were converted from mg/dL to mmol/L (mg/dL × 0.01129) before to LAP calculation.

### Multiplex cytokine and chemokine assay

Fasting blood samples were collected in EDTA tubes, placed on ice immediately, centrifuged at 4,000 rpm for 20 min at 4 °C, and the plasma was aliquoted and stored at − 80 °C until analysis. Cytokine and chemokine concentrations were determined using a multiplex ELISA-based immunoassay (MILLIPLEX MAP Human Cytokine/Chemokine Magnetic Bead Panel, Millipore, Burlington, MA, USA), according to the manufacturer’s instructions. The following analytes were measured: EGF, Eotaxin, G-CSF, GM-CSF, IFN-α2, IL-10, IL-12p40, IL-12p70, IL-13, IL-15, IL-17, IL-1Ra, IL-1α, IL-1β, IL-2, IL-3, IL-4, IL-5, IL-6, IL-7, IL-8, MCP-1, MIP-1α, MIP-1β, TNF-α, TNF-β, VEGF, and RANTES. All measurements were performed in duplicate. Concentrations were calculated from standard curves using Bio-Plex Manager Software (version 5, Bio-Rad Laboratories, Hercules, CA, USA).

### Statistical analysis

Continuous variables were expressed as mean ± standard deviation (SD). Group comparisons were performed using Student’s t-test for continuous variables and Pearson’s χ² test for categorical variables, as appropriate. Cytokine concentrations were log-transformed for analyses; results are presented on the log scale to ensure consistency with parametric testing. Simple and partial Pearson’s correlation coefficients were computed to assess relationships between cytokines, age, BMI, and LAP index. Multiple linear regression analyses were then performed to examine independent associations between LAP index and selected cytokines, adjusting progressively for potential confounders: Model 1: age, sex; Model 2: + BMI; Model 3: + IADL, CIRS-G, glycaemia, HDL-C. Sample size adequacy was evaluated with a post hoc power calculation for multiple linear regression (effect size 0.35, α = 0.05), yielding a statistical power of 99% (G*Power version 3.1.7). All tests were two-tailed, with *p* ≤ 0.05 considered statistically significant. Statistical analyses were conducted using IBM SPSS Statistics version 26 (IBM, Chicago, IL, USA).

## Results

### Study population and baseline characteristics

A total of 206 community-dwelling older adults were included (57.3% women, *n* = 118), with a mean age of 75.9 ± 7.5 years. Age distribution did not differ by sex (*p* = 0.919). Compared with men, women showed slightly lower functional independence in basic activities of daily living (BADL: 5.18 ± 1.08 vs. 5.48 ± 0.95; *p* = 0.041), higher depressive symptom burden (GDS: 4.83 ± 3.18 vs. 3.64 ± 2.48; *p* = 0.006), lower global cognitive performance (MMSE: 23.19 ± 6.27 vs. 26.28 ± 5.36; *p* < 0.0001), and lower nutritional status (MNA: 24.32 ± 2.85 vs. 25.62 ± 1.88; *p* = 0.002). No sex differences were observed for instrumental activities of daily living (IADL; *p* = 0.246).

As expected, anthropometric measures displayed marked sexual dimorphism: women had lower body weight and height (both *p* < 0.0001) and lower waist circumference and waist-to-hip ratio (both *p* < 0.0001), whereas BMI did not differ by sex (*p* = 0.699). The LAP index was comparable between women and men (42.8 ± 31.9 vs. 43.8 ± 29.3; *p* = 0.825). Women showed higher total cholesterol (211.7 ± 38.6 vs. 191.0 ± 41.1 mg/dL; *p* < 0.0001), LDL-C (124.3 ± 35.7 vs. 112.3 ± 34.5 mg/dL; *p* = 0.020), and HDL-C (63.7 ± 17.9 vs. 54.5 ± 14.4 mg/dL; *p* < 0.0001), while triglycerides were similar between sexes (*p* = 0.587). Finally, global comorbidity burden, assessed by CIRS-G, did not differ by sex (*p* = 0.575) (Table 1).

**Table 1:** Baseline clinical, anthropometric, and biochemical characteristics of the study population (*n* = 206)

Variable	Total (*n* = 206)	Women (*n* = 118)	Men (*n* = 88)	*p*
**Sociodemographic and functional**				
Age, years	75.9 ± 7.5	75.9 ± 8.0	76.0 ± 6.7	0.919
BADL, n	5.31 ± 1.04	5.18 ± 1.08	5.48 ± 0.95	0.041
IADL, n	5.31 ± 2.41	5.47 ± 2.62	5.08 ± 2.09	0.246
GDS, n	4.32 ± 2.95	4.83 ± 3.18	3.64 ± 2.48	0.006
MMSE, n	24.51 ± 6.08	23.19 ± 6.27	26.28 ± 5.36	< 0.0001
MNA, n	24.91 ± 2.54	24.32 ± 2.85	25.62 ± 1.88	0.002
**Anthropometric and metabolic indices**				
Weight, kg	68.4 ± 13.5	62.7 ± 11.4	76.1 ± 12.3	< 0.0001
Height, m	1.59 ± 0.09	1.53 ± 0.05	1.68 ± 0.06	< 0.0001
BMI, kg/m²	26.68 ± 4.22	26.58 ± 4.69	26.82 ± 3.53	0.699
Waist circumference, cm	91.2 ± 12.7	87.2 ± 13.4	96.6 ± 9.5	< 0.0001
Hip circumference, cm	103.3 ± 9.8	102.9 ± 11.2	103.9 ± 7.6	0.493
Waist-to-hip ratio	0.88 ± 0.08	0.84 ± 0.08	0.92 ± 0.06	< 0.0001
LAP index	43.2 ± 30.7	42.8 ± 31.9	43.8 ± 29.3	0.825
**Hematological and biochemical**				
RBC, ×10⁶/µL	4.57 ± 0.40	4.44 ± 0.42	4.75 ± 0.30	0.029
Platelets, ×10³/µL	221.1 ± 62.5	238.8 ± 58.6	198.1 ± 60.2	< 0.0001
Hemoglobin, g/dL	13.56 ± 1.06	13.12 ± 0.80	14.26 ± 1.07	< 0.0001
Glycaemia, mg/dL	102.7 ± 24.0	101.9 ± 25.7	103.7 ± 21.7	0.611
Uric acid, mg/dL	5.21 ± 1.35	4.82 ± 1.43	5.80 ± 1.02	0.043
**Lipid profile**				
Total cholesterol, mg/dL	202.9 ± 40.9	211.7 ± 38.6	191.0 ± 41.1	< 0.0001
LDL-C, mg/dL	119.2 ± 35.6	124.3 ± 35.7	112.3 ± 34.5	0.020
HDL-C, mg/dL	59.8 ± 17.1	63.7 ± 17.9	54.5 ± 14.4	< 0.0001
Triglycerides, mg/dL	118.8 ± 53.1	120.6 ± 51.0	116.5 ± 56.1	0.587
**Cardiovascular and comorbidity indices**				
Heart rate, bpm	64.8 ± 9.7	66.9 ± 10.1	61.9 ± 8.4	< 0.0001
Systolic BP, mmHg	128.0 ± 15.5	130.2 ± 16.5	124.8 ± 13.4	0.029
Diastolic BP, mmHg	73.1 ± 10.2	72.5 ± 10.6	74.0 ± 9.5	0.363
CIRS-G score, n	8.33 ± 4.60	8.49 ± 4.66	8.09 ± 4.53	0.575

Values are mean ± SD. Sex comparisons were performed using Student’s t-test for continuous variables. BADL, Basic Activities of Daily Living; IADL, Instrumental Activities of Daily Living; GDS, Geriatric Depression Scale; MMSE, Mini-Mental State Examination; MNA, Mini Nutritional Assessment; BMI, body mass index; LAP, lipid accumulation product; RBC, red blood cells; LDL-C, low-density lipoprotein cholesterol; HDL-C, high-density lipoprotein cholesterol; BP, blood pressure; CIRS-G, Cumulative Illness Rating Scale–Geriatrics

### Sex-related differences in circulating cytokines

On log-transformed concentrations, several circulating cytokines differed by sex. Women showed lower levels of IL-10 (*p* = 0.011), IL-12p70 (*p* = 0.004), IL-3 (*p* = 0.017), IL-4 (*p* = 0.006), and IL-15 (*p* = 0.013), whereas RANTES/CCL5 was modestly higher in women (*p* = 0.029). No other cytokines or growth factors showed sex-related differences (data not shown). Detailed values for molecules exhibiting significant sex differences are reported in Table 2.

**Table 2:** Plasma cytokines showing significant sex differences (log-transformed pg/mL; *n* = 206)

Molecule	Total (*n* = 206)	Women (*n* = 118)	Men (*n* = 88)	*p*
IL-3	−0.10 ± 0.31	−0.15 ± 0.29	−0.04 ± 0.31	0.017
IL-4	0.91 ± 0.94	0.76 ± 1.00	1.12 ± 0.80	0.006
IL-10	0.10 ± 0.88	−0.02 ± 0.95	0.28 ± 0.74	0.011
IL-12p70	0.71 ± 0.52	0.62 ± 0.58	0.83 ± 0.41	0.004
IL-15	0.26 ± 0.77	0.15 ± 0.86	0.42 ± 0.59	0.013
RANTES/CCL5	4.47 ± 0.34	4.52 ± 0.33	4.41 ± 0.35	0.029

Values are mean ± SD. Sex differences were tested with Student’s t-test on log-transformed concentrations. IL, interleukin; RANTES/CCL5, regulated upon Activation, Normal T Cell Expressed and Secreted

### Correlates of the LAP index in the overall cohort

Across the whole sample, the LAP index correlated positively with BMI (*r* = 0.682; *p* < 0.001), glycaemia (*r* = 0.321; *p* < 0.001) and with comorbidity burden (CIRS-G: *r* = 0.162; *p* = 0.033). LAP index was inversely correlated with HDL-C (*r* = − 0.246; *p* < 0.001) and with IADL (*r* = − 0.147; *p* = 0.035). No significant correlations were observed between LAP index and all other variables reported.

### Associations between immune mediators, age, adiposity, and LAP

**Fig. 1:**
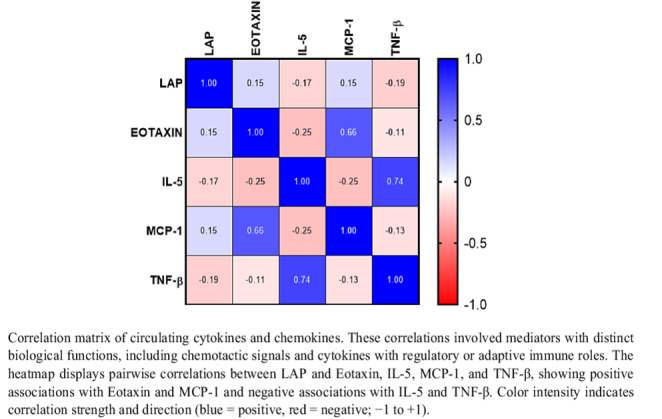
Heatmap of LAP index, Eotaxin, IL-5, MCP-1, and TNF-β

With increasing age, several immune mediators showed lower circulating concentrations, including IL-10, IL-12p40, IL-12p70, IL-13, IL-15, IL-17, IL-1RA, IL-2, IL-3, IL-4, IL-7, MIP-1α, and G-CSF (all *p* < 0.05). In contrast, TNF-α exhibited a modest positive association with age (*r* = 0.179; *p* = 0.010). Among all molecules analyzed, BMI was positively correlated with IL-6 (*r* = 0.172; *p* = 0.014) and TNF-α (*r* = 0.203; *p* = 0.004), and these relationships remained after adjustment for age and sex. Regarding the primary exposure, the LAP index correlated positively with eotaxin (*r* = 0.152; *p* = 0.029) and MCP-1 (*r* = 0.145; *p* = 0.038), and inversely with IL-5 (*r* = − 0.169; *p* = 0.016) and TNF-β (*r* = − 0.189; *p* = 0.007). After adjustment for age and sex, all four correlations remained significant: eotaxin (*r* = 0.149; *p* = 0.033), MCP-1 (*r* = 0.141; *p* = 0.040), IL-5 (*r* = − 0.167; *p* = 0.018), and TNF-β (*r* = − 0.185; *p* = 0.009) (Fig. 1).

### Multivariable determinants of the LAP index

Multivariable linear regression models were used to examine whether immune mediators associated with LAP remained independently related after accounting for clinically relevant covariates. In models adjusted for age and sex, eotaxin showed a positive association with LAP (B = 19.436; *p* = 0.033). This association remained significant after additional adjustment for BMI (B = 14.767; *p* = 0.027) but was attenuated in the fully adjusted model including IADL, CIRS-G, glycaemia, and HDL-C (B = 9.336; *p* = 0.229). For MCP-1, the association with LAP was significant in the age- and sex-adjusted model (B = 20.425; *p* = 0.044) but became non-significant after inclusion of BMI (B = 8.132; *p* = 0.278) and in the fully adjusted model (B = − 0.685; *p* = 0.936) IL-5 showed a consistent inverse association with LAP. The relationship was evident in the age- and sex-adjusted model (B = − 7.123; *p* = 0.018) and remained significant after adjustment for BMI (B = − 6.086; *p* = 0.006) and in the fully adjusted model (B = − 5.587; *p* = 0.023) (Table 3). TNF-β showed a robust inverse association with LAP. The relationship was significant in age- and sex-adjusted models (B = − 9.811; *p* = 0.009), strengthened after adjustment for BMI (B = − 10.838; *p* < 0.001), and remained significant in the fully adjusted model (B = − 9.371; *p* = 0.002), indicating an association independent of BMI, functional status, comorbidity burden, glycaemia, and HDL-C (Table 4).

**Table 3:** Association between IL-5 and LAP index (linear regression; *n* = 206)

Variable	B	β	95% CI	*p*
**Model 1 (age- and sex-adjusted)**				
Sex	−1.511	−0.024	−10.109 to 7.086	0.729
Age, years	0.103	0.025	−0.466 to 0.671	0.722
IL-5	−7.123	−0.168	−12.995 to − 1.250	0.018
R²	0.030			
**Model 2 (+ BMI)**				
Sex	−0.345	−0.006	−6.603 to 5.913	0.913
Age, years	0.202	0.049	−0.212 to 0.615	0.337
IL-5	−6.086	−0.144	−10.362 to − 1.810	0.006
BMI, kg/m²	4.931	0.679	4.202 to 5.660	< 0.001
R²	0.489			
**Model 3 (fully adjusted)**				
Sex	1.433	0.023	−5.839 to 8.706	0.698
Age, years	0.047	0.010	−0.492 to 0.585	0.864
IL-5	−5.587	−0.128	−10.410 to − 0.763	0.023
BMI, kg/m²	4.550	0.623	3.717 to 5.384	< 0.001
IADL, n	−0.771	−0.059	−2.320 to 0.777	0.327
CIRS-G, n	−0.146	−0.022	−0.921 to 0.630	0.711
Glycaemia, mg/dL	0.209	0.162	0.062 to 0.356	0.006
HDL-C, mg/dL	−0.200	−0.102	−0.432 to 0.032	0.091
R²	0.551			

Linear regression with LAP index as dependent variable. Sex coded as male = 0, female = 1

**Table 4:** Association between TNF-β and LAP index (linear regression; *n* = 206)

Variable	B	β	95% CI	*p*
**Model 1 (age- and sex-adjusted)**				
Sex	−0.382	−0.006	−8.946 to 8.181	0.930
Age, years	0.079	0.027	−0.490 to 0.647	0.786
TNF-β	−9.811	−0.186	−17.101 to − 2.521	0.009
R²	0.036			
**Model 2 (+ BMI)**				
Sex	0.579	0.009	−5.530 to 6.688	0.852
Age, years	0.148	0.036	−0.258 to 0.553	0.473
TNF-β	−10.838	−0.206	−16.040 to − 5.637	< 0.001
BMI, kg/m²	5.029	0.691	4.315 to 5.742	< 0.001
R²	0.512			
**Model 3 (fully adjusted)**				
Sex	2.490	0.039	−4.600 to 9.581	0.489
Age, years	0.030	0.007	−0.497 to 0.557	0.910
TNF-β	−9.371	−0.169	−15.337 to − 3.405	0.002
BMI, kg/m²	4.608	0.414	3.790 to 5.425	< 0.001
IADL, n	−0.751	−0.058	−2.257 to 0.756	0.327
CIRS-G, n	−0.136	−0.020	−0.898 to 0.627	0.725
Glycaemia, mg/dL	0.191	0.149	0.047 to 0.334	0.010
HDL-C, mg/dL	−0.196	−0.102	−0.420 to 0.027	0.085
R²	0.733			

Linear regression with LAP index as dependent variable. Sex coded as male = 0, female = 1

Indeed, to assess whether LAP-related immune associations were driven by abdominal adiposity, additional analyses were performed using waist-to-hip ratio with the same multivariable adjustments applied to LAP. IL-5 was inversely associated with waist-to-hip ratio after full adjustment (R²=0.412), whereas the association between TNF-β and waist-to-hip ratio was attenuated and no longer significant in fully adjusted models (Supplementary Tables S1–S2).

## Discussion

Traditional anthropometric indices such as BMI provide only a partial representation of metabolic health in older adults and may inadequately capture the immunological burden associated with adipose tissue expansion in late life [10]. Aging is characterized by profound changes in body composition, including loss of lean mass and preferential accumulation of visceral adiposity [25], which exerts disproportionate metabolic and immune effects often independent of body weight [26]. In this context, the LAP, integrating waist circumference and circulating triglycerides, represents a more specific surrogate of visceral adiposity and its metabolic–immune consequences [14]. In our cohort of community-dwelling older adults, LAP—but not BMI—was consistently associated with distinct immune signatures, supporting the concept that LAP captures biologically relevant immunometabolic information. Complementary analyses using waist-to-hip ratio showed weaker and less consistent immune associations, further reinforcing the added biological value of LAP.

According to previous literature [27, 28], BMI in our study is primarily associated with classical pro-inflammatory cytokines such as IL-6 and TNF-α, confirming its relationship with adipose-derived innate inflammatory activation [27]. These correlations remained significant after adjustment for age and sex, reflecting the well-established link between overall adiposity and low-grade inflammation [29]. In contrast, visceral adiposity as assessed by LAP is associated with a more heterogeneous immune profile, extending beyond canonical pro-inflammatory pathways. Chemokines such as MCP-1 and eotaxin, which regulate immune cell recruitment [30] and tissue infiltration [31], show positive associations with LAP, while cytokines involved in immune regulation and adaptive immunity, namely IL-5 and TNF-β, are inversely associated with LAP.

The positive associations between LAP and MCP-1 and eotaxin are consistent with adipose tissue–driven inflammatory cell recruitment within visceral fat depots [32, 33]. However, the attenuation of the MCP-1–LAP association after adjustment for BMI suggests that this pathway largely reflects overall adiposity burden, in line with the established role of MCP-1 in macrophage recruitment [34] and adipose inflammation [25]. These findings indicate that chemokine-driven inflammation may represent a canonical component of adiposity-related immune activation and is substantially shared across different adiposity measures.

In contrast, the inverse associations between LAP and IL-5 and TNF-β persist after adjustment for BMI and additional clinically relevant covariates, including functional status, comorbidity burden, glycaemia, and HDL cholesterol. Importantly, when waist-to-hip ratio is modeled using the same multivariable strategy, its associations with IL-5 and TNF-β are attenuated after inclusion of BMI, reflecting collinearity with overall adiposity. The persistence of IL-5 and TNF-β associations with LAP despite BMI adjustment indicates that LAP captures immunometabolic information beyond adiposity quantity alone, likely related to the combined lipid–anthropometric construct underlying visceral fat accumulation. From a statistical standpoint, the stability of the IL-5 and TNF-β coefficients across sequential models supports an association independent of measured confounders.

IL-5 is a Th2-associated cytokine involved in eosinophil activation and immune homeostasis and has been increasingly recognized as a regulator of adipose tissue immune balance [35, 36]. Beyond its canonical role in eosinophil biology, IL-5–dependent immune circuits support alternatively activated macrophage polarization and contribute to the maintenance of adipose tissue immune homeostasis. The independent inverse association between LAP and IL-5 observed in our study may therefore reflect a loss of protective or regulatory immune signaling rather than simple attenuation of inflammation. Although speculative, this interpretation is biologically plausible and suggests that increasing visceral adiposity in older adults may be accompanied by alteration of immune resilience and diminished capacity to preserve immune homeostasis within adipose tissue. Similarly, TNF-β (lymphotoxin-α) is biologically distinct from TNF-α and should not be interpreted as a classical adipose-derived pro-inflammatory cytokine [37]. TNF-β is primarily produced by activated lymphocytes and plays a central role in adaptive immunity, lymphoid tissue organization, and maintenance of lymphoid microenvironments [38, 39]. The robust inverse association between TNF-β and LAP—independent of BMI—suggests impaired lymphocyte-related signaling and erosion of adaptive immune competence in individuals with higher visceral adiposity, consistent with emerging evidence linking lymphotoxin signaling to immunometabolic regulation [40]. Within the framework of aging, these findings align with contemporary models of immunosenescence, in which inflammaging reflects immune imbalance rather than generalized upregulation of inflammatory mediators [41]. Increased visceral adiposity may therefore contribute not only to chemokine-driven innate inflammation but also to qualitative deterioration of regulatory and adaptive immune pathways. This selective immune remodeling provides a more innovative interpretation of inflammaging and highlights the importance of considering visceral adiposity–specific immune signatures in older adults.

From a clinical perspective, LAP index is a simple and inexpensive measure readily applicable in geriatric and epidemiological settings. Its ability to capture visceral adiposity–specific immune signatures may help identify older individuals at increased immunometabolic vulnerability with potential implications for risk stratification and targeted preventive strategies.

The strengths of this study include the well-characterized cohort, the simultaneous assessment of a large immune mediator panel, and the use of multivariable models to disentangle adiposity-mediated from adiposity-independent associations. Nevertheless, the cross-sectional design precludes causal inference, and single-time-point cytokine measurements may not fully capture dynamic immune regulation. The absence of imaging-based measures of visceral fat, hormonal data, and detailed lifestyle factors also limits mechanistic interpretation, and residual confounding cannot be excluded.

In conclusion, this study identifies a potential distinct immunometabolic signature of visceral adiposity in older adults, characterized by BMI-independent inverse associations of IL-5 and TNF-β with the LAP index. These findings underscore the relevance of visceral fat as a driver of immune dysregulation during aging and support the use of LAP as a clinically meaningful marker beyond BMI. Longitudinal and mechanistic studies are warranted to clarify causal pathways and to determine whether modulation of these immune signals may attenuate inflammaging and improve resilience in later life.

## Supplementary Information

Below is the link to the electronic supplementary material.

Supplementary Material 1

## Data Availability

No datasets were generated or analysed during the current study.
