# Supplementary material for: Beyond BMI: visceral adiposity assessed by the lipid accumulation product index shows independent associations with IL-5 and TNF-β in older adults
Source: Aging Clin Exp Res. 2026 Feb 24;38(1):93. doi: 10.1007/s40520-026-03338-y (PMC12979275; doi:10.1007/s40520-026-03338-y)
Supplement: Supplementary file 1 — Supplementary Material 1 [file 40520_2026_3338_MOESM1_ESM.docx]

**Supplementary material**

**Table S1. Association between IL-5 and waist circumference (linear regression; n = 206)**

|  | **B** | **β** | **95% CI** | **p** |
| --- | --- | --- | --- | --- |
| **Model 1 (age- and sex-adjusted + BMI)** |  |  |  |  |
| Sex (female = 1) | −9.212 | −0.359 | −11.060 to −7.363 | <0.001 |
| Age, years | 0.070 | 0.041 | −0.052 to 0.192 | 0.262 |
| IL-5 | −2.896 | −0.166 | −4.159 to −1.634 | <0.001 |
| BMI, kg/m² | 2.265 | 0.755 | 2.050 to 2.480 | <0.001 |
| R² |  |  |  | 0.738 |
| **Model 2 (fully adjusted)** |  |  |  |  |
| Sex (female = 1) | −9.121 | −0.361 | −11.263 to −6.979 | <0.001 |
| Age, years | −0.014 | −0.008 | −0.173 to 0.144 | 0.861 |
| IL-5 | −2.714 | −0.719 | −4.135 to −1.293 | <0.001 |
| BMI, kg/m² | 2.104 | 0.124 | 1.859 to 2.350 | <0.001 |
| IADL, n | −0.285 | −0.055 | −0.741 to 0.171 | 0.218 |
| CIRS-G, n | 0.221 | 0.083 | −0.007 to 0.450 | 0.057 |
| Glycaemia, mg/dL | 0.041 | 0.080 | −0.002 to 0.084 | 0.064 |
| HDL-C, mg/dL | −0.026 | −0.033 | −0.094 to 0.043 | 0.460 |
| R² |  |  |  | 0.740 |

**Table S2. Association between TNF-β and waist circumference (linear regression; n = 206)**

|  | **B** | **β** | **95% CI** | **p** |
| --- | --- | --- | --- | --- |
| **Model 1 (age- and sex-adjusted + BMI)** |  |  |  |  |
| Sex (female = 1) | −8.796 | −0.343 | −10.627 to −6.965 | <0.001 |
| Age, years | 0.061 | 0.036 | −0.061 to 0.182 | 0.326 |
| TNF-β | −3.873 | −0.179 | −5.432 to −2.314 | <0.001 |
| BMI, kg/m² | 2.292 | 0.765 | 2.079 to 2.506 | <0.001 |
| R² |  |  |  | 0.714 |
| **Model 2 (fully adjusted)** |  |  |  |  |
| Sex (female = 1) | −8.747 | −0.345 | −10.872 to −6.622 | <0.001 |
| Age, years | −0.026 | −0.014 | −0.184 to 0.131 | 0.742 |
| TNF-β | −3.344 | −0.151 | −5.132 to −1.556 | <0.001 |
| BMI, kg/m² | 2.129 | 0.730 | 1.884 to 2.374 | <0.001 |
| IADL, n | −0.331 | −0.060 | −0.762 to 0.141 | 0.176 |
| CIRS-G, n | 0.240 | 0.089 | 0.012 to 0.469 | 0.039 |
| Glycaemia, mg/dL | 0.035 | 0.068 | −0.008 to 0.078 | 0.110 |
| HDL-C, mg/dL | −0.020 | −0.025 | −0.087 to 0.047 | 0.565 |
| R² |  |  |  | 0.740 |

**Table S3. Association between IL-5 and waist-to-hip ratio (linear regression; n = 206)**

|  | **B** | **β** | | **95% CI** | | **p** | |
| --- | --- | --- | --- | --- | --- | --- | --- |
| **Model 1 (age- and sex-adjusted + BMI)** |  |  | |  | |  | |
| Sex (female = 1) | −0.088 | −0.526 | | −0.106 to −0.070 | | <0.001 | |
| Age, years | 0.001 | 0.047 | | −0.001 to 0.002 | | 0.387 | |
| IL-5 | −0.020 | −0.177 | | −0.032 to −0.008 | | 0.002 | |
| BMI, kg/m² | 0.006 | 0.325 | | 0.004 to 0.008 | | <0.001 | |
| R² |  |  | |  | | 0.413 | |
| **Model 2 (fully adjusted)** |  |  | |  | |  | |
| Sex (female = 1) | −0.081 | −0.489 | | −0.102 to −0.060 | | <0.001 | |
| Age, years | 0.000 | −0.011 | | −0.002 to 0.001 | | 0.869 | |
| IL-5 | −0.015 | −0.134 | | −0.029 to −0.001 | | 0.032 | |
| BMI, kg/m² | 0.005 | 0.257 | | 0.002 to 0.007 | | <0.001 | |
| IADL, n | −0.003 | −0.080 | | −0.007 to 0.002 | | 0.233 | |
| CIRS-G, n | 0.003 | 0.151 | | 0.000 to 0.005 | | 0.057 | |
| Glycaemia, mg/dL | 9.609 | 0.029 | | 0.000 to 0.001 | | 0.656 | |
| HDL-C, mg/dL | −0.001 | −0.100 | | −0.001 to 0.000 | | 0.139 | |
| R² |  |  |  | | 0.412 | |  |

**Table S4. Association between TNF-β and waist-to-hip ratio (linear regression; n = 206)**

|  | **B** | **β** | **95% CI** | **p** |
| --- | --- | --- | --- | --- |
| **Model 1 (age- and sex-adjusted + BMI)** |  |  |  |  |
| Sex (female = 1) | −0.081 | −0.462 | −0.100 to −0.061 | <0.001 |
| Age, years | 0.000 | 0.029 | −0.001 to 0.002 | 0.631 |
| TNF-β | −0.027 | −0.184 | −0.044 to −0.010 | 0.002 |
| BMI, kg/m² | 0.006 | 0.300 | 0.004 to 0.008 | <0.001 |
| R² |  |  |  | 0.344 |
| **Model 2 (fully adjusted)** |  |  |  |  |
| Sex (female = 1) | −0.070 | −0.402 | −0.093 to −0.047 | <0.001 |
| Age, years | −0.001 | −0.041 | −0.002 to 0.001 | 0.560 |
| TNF-β | −0.019 | −0.124 | −0.038 to 0.001 | 0.058 |
| BMI, kg/m² | 0.004 | 0.220 | 0.002 to 0.007 | 0.001 |
| IADL, n | −0.003 | −0.096 | −0.008 to 0.001 | 0.170 |
| CIRS-G, n | 0.003 | 0.137 | 0.000 to 0.005 | 0.045 |
| Glycaemia, mg/dL | −3.979 | −0.011 | −0.001 to 0.000 | 0.867 |
| HDL-C, mg/dL | −0.001 | −0.161 | −0.0002 to 0.000 | 0.022 |
| R² |  |  |  | 0.348 |

Linear regression with waist circumference (Tables S1–S2) or waist-to-hip ratio (Tables S3–S4) as the dependent variable. Sex coded as male = 0, female = 1. BMI, body mass index; IADL, Instrumental Activities of Daily Living; CIRS-G, Cumulative Illness Rating Scale–Geriatrics; HDL-C, high-density lipoprotein cholesterol
